# Comparative secretome analysis unveils species-specific virulence factors in *Elsinoe perseae*, the causative agent of the scab disease of avocado (*Persea americana*)

**DOI:** 10.3934/microbiol.2024039

**Published:** 2024-10-28

**Authors:** Biju Vadakkemukadiyil Chellappan

**Affiliations:** Department of Biological Sciences, College of Science, King Faisal University, P.O. Box 420, Al-Ahsa 31982, Saudi Arabia

**Keywords:** CAZymes, cell wall–degrading enzymes, effector, *Elsinoe perseae*, proteases, secretome

## Abstract

The scab disease, caused by *Elsinoe perseae*, poses a significant risk to avocado (*Persea americana*) production in countries with warm and humid climates. Although the genome has been published, the precise virulence factors accountable for the pathogenicity of *E. perseae* have not yet been determined. The current study employed an in silico approach to identify and functionally characterize the secretory proteins of *E. perseae*. A total of 654 potential secretory proteins were identified, of which 190 were classified as carbohydrate-active enzymes (CAZymes), 49 as proteases, and 155 as potential effectors. A comparison to six other closely related species identified 40 species-specific putative effectors in *E. perseae*, indicating their specific involvement in the pathogenicity of *E. perseae* on avocado. The data presented in this study might be valuable for further research focused on understanding the molecular mechanisms that contribute to the pathogenicity of *E. perseae* on avocado.

## 1. Introduction

Fungal infections are responsible for significant reductions in agricultural crop yields and post-harvest product losses on a global scale [1]. Approximately $220 billion is wasted annually in the worldwide economy due to fungal diseases (FAO, 2019). In order to mitigate such losses, farmers employ multiple fungicides, a practice that proves to be ineffective due to the rapid development of disease resistance to these chemicals. Furthermore, this method presents substantial hazards to both human well-being and the ecosystem. In contrast, genetic approaches, such as the integration of resistant genes, are more reliable and enduring. Nevertheless, the existence of specific genes that confer resistance in host plants can exert substantial selective pressure on pathogens, leading to rapid evolutionary adaptations that often give rise to new races capable of evading host resistance, as observed in numerous interactions between plants and pathogens [2],[3]. Hence, to attain enhanced and long-lasting protection against fungal diseases, it is imperative to possess a profound comprehension of the pathogenicity factors secreted by the pathogen and the subsequent resistance responses of the plant [4].

To effectively invade the host plant, plant-pathogenic fungi release several proteins to overcome various host protection barriers. These secretory proteins contain several enzymes that break down the cell wall, proteases, and small secretory proteins known as "effectors". Multiple investigations on plant–microbe interactions have demonstrated that effectors significantly influence the pathogenicity of numerous phytopathogens [5],[6]. Effectors are small proteins that pathogens release into both the extracellular and intracellular regions of host plants with the purpose of modifying specific targets inside the host. They exhibit distinct attributes, including small size (50–300 amino acids), high cysteine content, possession of an N-terminal signal peptide, absence of transmembrane structural domains, glycosylphosphatidylinositol (GPI) anchor sites, and subcellular localization signals for chloroplasts or other intracellular organelles [7]. These inherent traits allow scientists to predict effectors from the genomes of numerous phytopathogens. For instance, in a recent study, the draft genome was utilized to forecast the potential effectors of the citrus pathogen, *Elsinoe fawcettii* [8]–[13].

Avocado scab, a prevalent disease in avocado-producing regions with warm and humid climates, is mostly caused by the fungus *E. perseae* [14],[15]. The initial symptoms manifest as distinct spots on the leaves along the midrib, which have the potential to combine and form star-shaped patterns. As the disease advances, the leaves undergo distortion and stunting [16]. The symptoms of avocado scab on fruits manifest as blackish/brownish oval and elevated cork scab formations that are dispersed on the fruit's skin [16],[17]. Even though it does not directly damage the pulp, it negatively affects the quality of the fruit and makes it inappropriate for the global market. This reduces the production value by up to 60%. To date, avocado disease has been reported in the USA and many countries in Africa and Asia [18].

Current disease management strategies for avocado scab are generally based on a combination of cultural and chemical methods. A crucial cultural practice involves the elimination and destruction of affected plant material to avert the dissemination of the disease [19]. In the chemical method, many fungicides, including benomyl, azoxystrobin, and pyraclostrobin, have shown efficiency in controlling the disease's spread [20],[21]. Nonetheless, overdependence on chemical pesticides may result in environmental issues and the emergence of fungicide resistance, hence requiring the investigation of alternate approaches. Biocontrol techniques remain inadequately researched, despite investigations conducted to manage other avocado pathogens, such as *Phytophthora cinnamomi* [22],[23]. Currently, there has been no molecular analysis, including gene expression studies, concerning the *E. perseae*–avocado interaction, conducted to identify the pathogenicity factors of *E. perseae* that increase its virulence on avocado.

To effectively mitigate the economic impact of avocado scab, a thorough comprehension of the molecular mechanisms underlying the pathogenicity of *E. perseae* is necessary [24],[25]. Although the draft genome of *E. perseae* is publicly available, there has been a dearth of comprehensive investigation about the exact characteristics and functionalities of its secretory proteins [24]. This study utilized a bioinformatics methodology to systematically predict secretory proteins in the genome of *E. perseae*. It provides valuable insights into numerous potential virulence factors of *E. perseae*, including cell wall–degrading enzymes, proteases, pathogenicity-related proteins, and potential effector proteins. This will serve as a great resource for future investigations into the molecular interaction between *E. perseae* and avocado.

## 2. Materials and methods

### 2.1. Sequence information and gene prediction

The *E. perseae* draft genome sequence (NCBI accession: GCA_029448695.1) was obtained from NCBI, and gene models were predicted in the current study using the GenSAS server [24],[25]. In this study, both homology and de novo methods were employed to predict gene models in the genome of *E. perseae*. In the homology-based prediction, the repeat-masked assembly was subjected to a BlastN search against expressed sequence tag (EST) sequences of the closely related species, *E. ampelina*, using an e-value cutoff of 1 × 10^−5^ [26],[27]. The de novo approach employed two *ab initio* gene prediction tools, namely Augustus and GeneMarkES [28],[29]. Ultimately, the gene prediction information was consolidated using EvidenceModeler to produce a non-redundant gene set [30]. The comparative analysis utilized genome data from six closely related species, viz., *E. necatrix*, *E. batatas*, *E. arachidis*, *E. fawcettii*, *E. ampelina*, and *E. australis* (Table 1) [31]–[35].

### 2.2. Prediction of the secretome

The previously outlined pipeline was employed to predict the fungal secretome [36]. To identify the sequences with signal peptide, SignalP (version 6.0) was employed in conjunction with the Phobius server [37],[38]. The sequences that were identified as having a signal peptide by both systems were chosen for additional screening. The DeepTMHMM server was employed to eliminate the transmembrane proteins [39]. The proteins targeting the endoplasmic reticulum (ER) were eliminated by scanning them for PS00014 ER motif retention using the Prosite database and the ScanProsite web service [40]. The proteins targeting various organelles such as mitochondria and chloroplast were predicted using the TargetP and WoLF PSORT systems [41],[42]. The sequences containing glycophosphatidylinositol (GPI) anchor motifs were identified using NetGPI (version 1.1) [43].

### 2.3. Characterization of secretome

The refined secretome was subjected to scanning against NCBI, InterPro, and PFAM databases to get functional annotations for the predicted proteins [44],[45]. The annotation of carbohydrate-active enzymes was retrieved using the CAZy database and dbCAN web server [46],[47]. The effector prediction was performed using EffectorP CAZy (version 3.0) software, in conjunction with manual examination [48]. Furthermore, the BlastP algorithm with a significance threshold of E value lower than 1 × 10^−10^ was employed to query the pathogen–host interaction database (PHI database) in order to identify any resemblances to established effectors and pathogenicity factors [49]. The identification of proteolytic enzymes was accomplished by a BlastP search conducted on the MEROPS database [50]. Orthologue analysis was conducted using Orthovenn3 web server [51]. To infer the phylogenetic relationship among *Elsinoe* species, 50 shared orthologs were selected randomly and a concatenated alignment was made. The relationship was constructed by MEGA11 using the maximum likelihood method and JTT matrix-based model (based on 1000 bootstrap replications).

## 3. Results and discussion

### 3.1. Secretome of Elsinoe perseae

**Figure 1.**
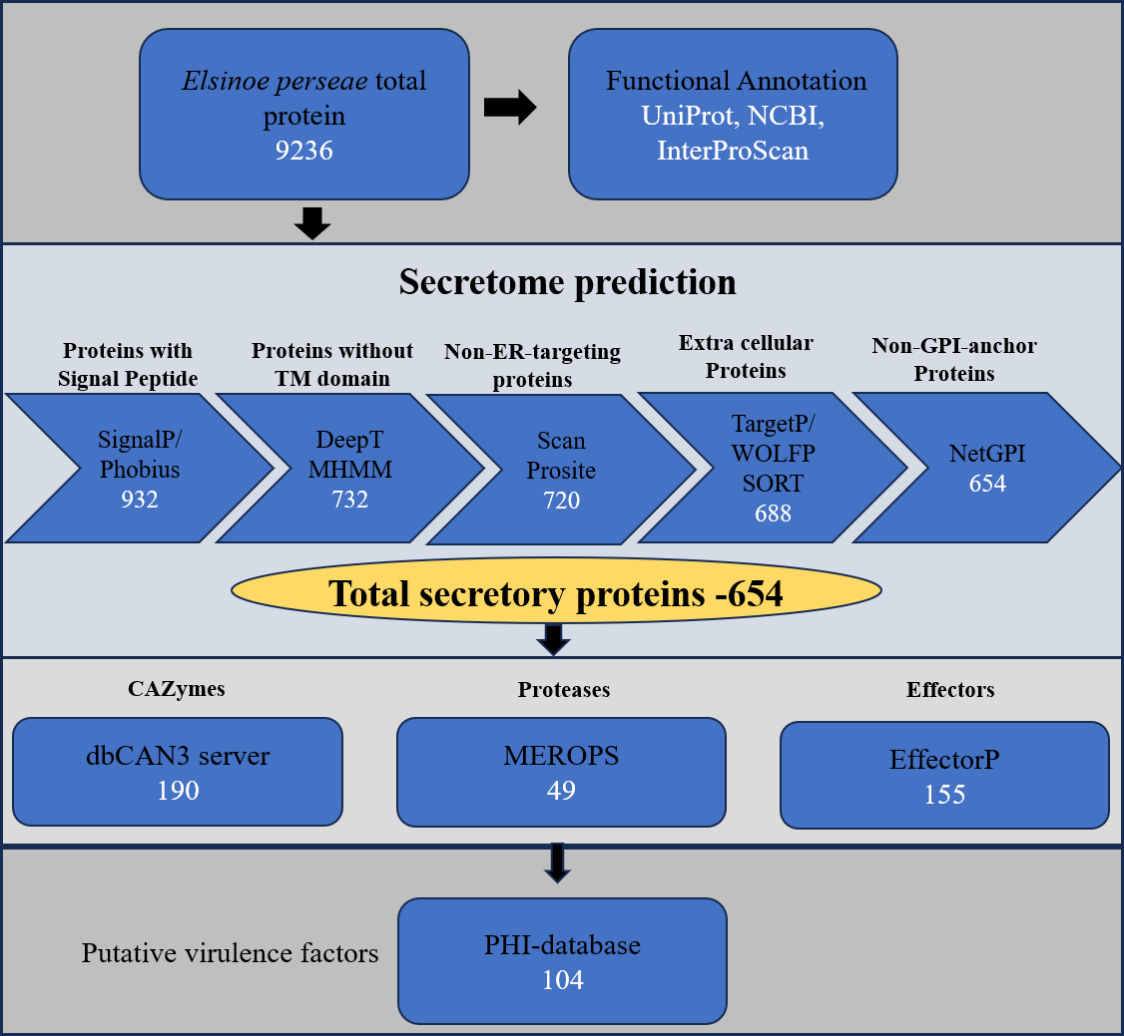
Pipeline for the identification and functional characterization of the secretome of *Elsinoe perseae*. CAZyme: carbohydrate-active enzymes; PHI: pathogen–host interactions database. Tools and the number of filtered proteins in each step are shown in the respective boxes.

The draft genome sequence of *E. perseae* (NCBI accession: GCA_029448695.1) was utilized to identify the secretome [24]. Furthermore, for the comparative study, the genome data of six *Elsinoe* species, namely *E. necatrix*, *E. batatas*, *E. arachidis*, *E. fawcettii*, *E. ampelina*, and *E. australis*, was chosen (Table 1) [31]–[35]. The gene models for *E. perseae*, *E. necatrix*, *E. batatas*, and *E. arachidis* were predicted in the current study using the GenSAS server due to the lack of publicly available annotations (Table 1, Table S1). Gene models for the remaining three species were acquired from publicly available databases (Table 1). In total, 9236 proteins were predicted from the *E. perseae* genome, which were subjected to secretome prediction using the methodology depicted in Figure 1. Of the 9236 proteins, an N-terminal secretory signal sequence was identified in 932 proteins. Out of these, 732 proteins, lacking any transmembrane domain, were chosen and examined for an ER-targeting signal in order to eliminate the proteins that reside in the endoplasmic reticulum. Of the 732 proteins, a subset of 12 were identified to possess the PS00014 ER motif and were therefore removed from subsequent research. Through the analysis of TargetP and WoLF PSORT, it was projected that the remaining 720 proteins were localized in the extracellular space. Out of the total 720 proteins, 66 proteins were identified as having GPI-anchor motifs using NetGPI (version 1.1). These proteins are most likely found on the surface rather than being secreted, and hence they were not included in the analysis. As a result, a list of 654 "refined secretome" was obtained, representing 7.1% of the entire predicted proteome of *E. perseae* (Figure 1, Table 1).

**Table 1.** List of *Elsinoe* species included in this study.

Species	Genome size (Mb)	GenBank assembly	Gene models	Secretome
*E. perseae*	23.5	GCA_029448695.1*	9236	654
*E. necatrix*	25.5	GCA_033846785.1*	8501	619
*E. batatas*	26.5	GCA_017309325.2*	8783	569
*E. arachidis*	33.2	GCA_013372555.1*	9754	781
*E. fawcettii*	26.3	GCA_007556565.1	10264	658
*E. ampelina*	28.3	GCA_005959805.1	10209	621
*E. australis*	23.8	GCA_007556505.1	9223	676

*Gene models predicted in this study were used for the analysis.

### 3.2. Structural and functional characterization of *E. perseae* secretome

**Figure 2.**
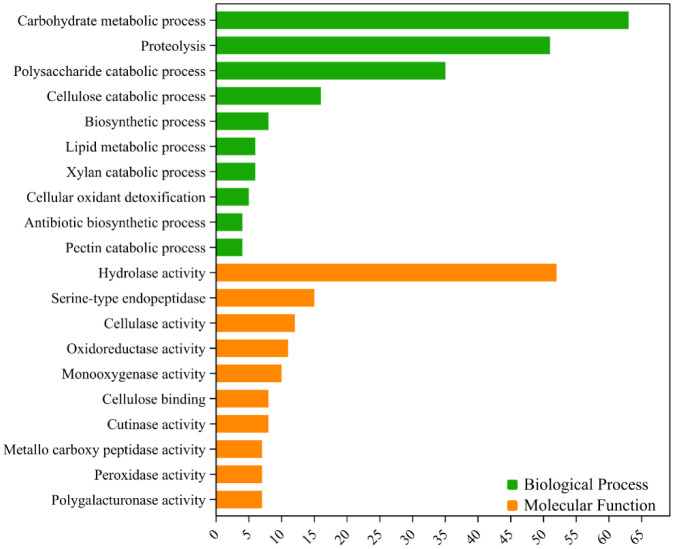
Gene ontology of the secretome of *Elsinoe perseae*. GO terms assigned to molecular function and biological processes are listed.

The refined secretome of *E. perseae* varied in length, ranging from 55 to 2410 amino acids (aa). Among these proteins, 50.3% (330) had a length of 55–399 aa, indicating an abundance of small secretory proteins in the secretome of *E. perseae* (Figure S1). Secretory proteins exhibited a molecular weight (MW) range of 5.9–248 kDa. The majority of these proteins (61.3%) fell between the 5.9 and 49.9 kDa range (Figure S1B). Furthermore, the theoretical isoelectric point (pI) of the secretory proteins varied between 3.45 and 11.89. The majority (53.3%) of these proteins had a pI ranging from 4 to 5.9 (Figure S1C). The domain analysis identified a minimum of one functional domain in 445 proteins. The most enriched domains were PAN_4 (PF14295), PAN_1 (PF00024), WSC (PF01822), LysM (PF01476), and FAD binding (PF00890) (Figure S3). From the total secreted proteins, 358 (55%) proteins were assigned at least one gene ontology (GO) term based on sequence homology. Based on the gene ontology terms, these proteins were categorized into three categories: biological process (251 proteins, 38.3%), molecular function (301 proteins, 46%), and cellular components (197 proteins, 30.1%) (Figure 2). The gene ontology terms that are most enriched under biological processes are carbohydrate metabolic process (GO:0005975), proteolysis (GO:0006508), polysaccharide catabolic process (GO:0000272), cellulose catabolic process (GO:0030245), and others (Figure 2). The molecular function category prominently encompasses several activities such as hydrolase activity (GO:0004553), serine-type endopeptidase activity (GO:0004252), cellulase activity (GO:0008810), or oxidoreductase activity (GO:0016614) (Figure 2). The cellular component comprises the extracellular region (GO:0005576), cell wall (GO:0005618), and membrane (GO:0016020) (not shown here).

**Figure 3.**
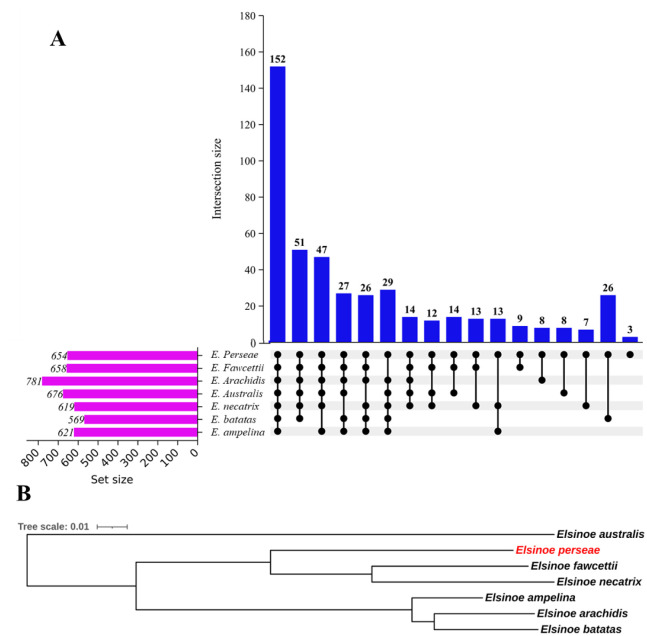
Comparative secretome and phylogenetic analysis of the secretome of seven *Elsinoe* species. A. Orthologue clusters. Clusters of orthologous genes were detected utilizing Orthovenn3 and displayed utilizing UpsetR. For the analysis, the e-value cutoff of 1 × 10^−10^ was utilized. Blue bars represent the number of clusters shared by *Elsinoe* species, represented by numbers shown above the bar. Line and dot connections between species that share orthologs. B. Phylogenetic relationship of seven species of *Elsinoe*. A concatenated alignment of fifty shared orthologue proteins was used to infer the phylogenetic tree of seven *Elsinoe* species. The relationship was constructed using the maximum likelihood method and JTT+CAT matrix-based model.

### 3.3. Secretome comparative and phylogenetic analysis

For the comparative analysis, the secretome of six closely related species of *Elsinoe*, as shown in Table 1, was identified using the methodology described above. The results showed that the secretome of *E. perseae* was comparable to that of closely related species, of which the closest was *E. arachidis* (781) and the furthest was *E. batatas* (569) (Table 1). The secretome of all species analyzed displayed the same pattern in terms of length, PI, and MW distribution (Figure S1A–C). The comparative orthologue analysis revealed that the secretome of all species of *Elsinoe* shared 152 clusters (Figure 3A). Among these clusters, 140 were identified as single-copy gene clusters. The analysis also identified singletons (proteins that do not form any clusters) in each species, which varied among species. *E. australis* had the maximum number of singletons (112), while *E. batatas* had the lowest (38) (Table S2). A total of 76 singletons were identified for *E. perseae*, indicating that 88.3% of the secretory proteins of *E. perseae* have orthologues in other species. A phylogenetic tree was constructed to elucidate the relationship between *Elsinoe* species using a composite alignment of 50 single-copy orthologue proteins (Figure 3B). Within the tree, *E. fawcettii* and *E. arachidis* showed a close relation with *E. necatrix* and *E. batatas*, respectively (Figure 3B). *E. perseae* constituted a distinct clade, indicating their monophyletic origin (Figure 3B), as previously shown [52].

**Table 2.** Cell wall–degrading enzymes in *E. perseae*.

CAZy family	Protein id	PFAM id	Enzyme name	Substrate
AA7	Ep.00g028650.m01	PF01565.18	Glucooligosaccharide oxidase	Cellobiose
AA7	Ep.00g057050.m01	PF01565.18	Glucooligosaccharide oxidase	Cellobiose
AA7	Ep.00g013270.m01	PF01565.18	Glucooligosaccharide oxidase	Cellobiose
AA7	Ep.00g035950.m01	PF01565.18	Glucooligosaccharide oxidase	Cellobiose
AA7	Ep.00g003880.m01	PF01565.18	Glucooligosaccharide oxidase	Cellobiose
AA7	Ep.00g009110.m01	PF01565.18	Glucooligosaccharide oxidase	Cellobiose
AA7	Ep.00g029870.m01	PF01565.18	Glucooligosaccharide oxidase	Cellobiose
AA7	Ep.00g005960.m01	PF01565.18	Glucooligosaccharide oxidase	Cellobiose
AA8	Ep.00g020830.m01	PF16010	Cellobiose dehydrogenase	Cellobiose
AA8	Ep.00g040980.m01	PF16010	Cellobiose dehydrogenase	Cellobiose
AA8	Ep.00g003740.m01	PF16010	Cellobiose dehydrogenase	Cellobiose
GH31	Ep.00g091100.m01	PF13802.1	α-glucosidase	Cellobiose
AA3	Ep.00g035640.m01	PF00732	Cellobiose dehydrogenases	Cellulose
AA3	Ep.00g090240.m01	PF00732	Cellobiose dehydrogenases	Cellulose
AA3	Ep.00g035790.m01	PF00732	Cellobiose dehydrogenases	Cellulose
AA9	Ep.00g003730.m01	PF03443	Lytic cellulose monooxygenase	Cellulose
AA9	Ep.00g012020.m01	PF03443	Lytic cellulose monooxygenase	Cellulose
AA9	Ep.00g019760.m01	PF03443	Lytic cellulose monooxygenase	Cellulose
AA9	Ep.00g021550.m01	PF03443	Lytic cellulose monooxygenase	Cellulose
AA9	Ep.00g027070.m01	PF03443	Lytic cellulose monooxygenase	Cellulose
AA9	Ep.00g028770.m01	PF03443	Lytic cellulose monooxygenase	Cellulose
AA9	Ep.00g030160.m01	PF03443	Lytic cellulose monooxygenase	Cellulose
AA9	Ep.00g056790.m01	PF03443	Lytic cellulose monooxygenase	Cellulose
AA9	Ep.00g058480.m01	PF03443	Lytic cellulose monooxygenase	Cellulose
AA9	Ep.00g061930.m01	PF03443	Lytic cellulose monooxygenase	Cellulose
AA9	Ep.00g067810.m01	PF03443	Lytic cellulose monooxygenase	Cellulose
AA9	Ep.00g072230.m01	PF03443	Lytic cellulose monooxygenase	Cellulose
AA9	Ep.00g078850.m01	PF03443	Lytic cellulose monooxygenase	Cellulose
AA9	Ep.00g083810.m01	PF03443	Lytic cellulose monooxygenase	Cellulose
AA9	Ep.00g089020.m01	PF03443	Lytic cellulose monooxygenase	Cellulose
AA16	Ep.00g004440.m01	PF03067	Lytic polysaccharide mono-oxygenase	Cellulose
AA16	Ep.00g076350.m01	PF03067	Lytic polysaccharide mono-oxygenase	Cellulose
AA16	Ep.00g091200.m01	PF03067	Lytic polysaccharide mono-oxygenase	Cellulose
GH1	Ep.00g078300.m01	PF00232.13	β-glucosidase	Cellulose
GH1	Ep.00g087300.m01	PF00232.13	β-glucosidase	Cellulose
GH3	Ep.00g040790.m01	PF00933.16	β-glucosidase	Cellulose
GH3	Ep.00g033660.m01	PF00933.16	β-glucosidase	Cellulose
GH3	Ep.00g062370.m01	PF00933.16	β-glucosidase	Cellulose
GH3	Ep.00g016320.m01	PF00933.16	β-glucosidase	Cellulose
GH3	Ep.00g005830.m01	PF00933.16	β-glucosidase	Cellulose
GH3	Ep.00g060380.m01	PF00933.16	β-glucosidase	Cellulose
GH3	Ep.00g019010.m01	PF00933.16	β-glucosidase	Cellulose
GH3	Ep.00g047030.m01	PF11220.3	β-glucosidase	Cellulose
GH39	Ep.00g072240.m01	PF01229.12	β-glucosidase	Cellulose
GH131	Ep.00g013780.m01	PF14099.1	β-glucosidase	Cellulose
GH128	Ep.00g075030.m01	PF18271.4	β-glucosidase	Cellulose
CE5	Ep.00g091760.m01	PF01083.17	Cutinase	Cutin
CE5	Ep.00g080690.m01	PF01083.17	Cutinase	Cutin
CE5	Ep.00g055890.m01	PF01083.17	Cutinase	Cutin
CE5	Ep.00g028100.m01	PF01083.17	Cutinase	Cutin
CE5	Ep.00g075580.m01	PF01083.17	Cutinase	Cutin
CE5	Ep.00g001590.m01	PF04131.9	Cutinase	Cutin
CE5	Ep.00g021220.m01	PF01083.17	Cutinase	Cutin
CE5	Ep.00g092920.m01	PF01083.17	Cutinase	Cutin
CE5	Ep.00g052640.m01	PF01083.17	Cutinase	Cutin
CE5	Ep.00g054680.m01	PF01083.17	Cutinase	Cutin
CE5	Ep.00g024250.m01	PF01083.17	Cutinase	Cutin
CE5	Ep.00g078410.m01	PF01083.17	Cutinase	Cutin
CE5	Ep.00g059400.m01	PF01083.17	Cutinase	Cutin
GH43	Ep.00g033080.m01	PF04616.9	Endo-α-1,5-L-arabinanase	Hemicellulose (arbinan)
GH43	Ep.00g079770.m01	PF04616.9	Endo-α-1,5-L-arabinanase	Hemicellulose (arbinan)
GH43	Ep.00g048420.m01	PF04616.9	Endo-α-1,5-L-arabinanase	Hemicellulose (arbinan)
GH43	Ep.00g013710.m01	PF04616.9	Endo-α-1,5-L-arabinanase	Hemicellulose (arbinan)
GH43	Ep.00g006660.m01	PF04616.9	Endo-α-1,5-L-arabinanase	Hemicellulose (arbinan)
GH43	Ep.00g014420.m01	PF00251.15	Endo-α-1,5-L-arabinanase	Hemicellulose (arbinan)
GH43	Ep.00g057540.m01	PF04616.9	Endo-α-1,5-L-arabinanase	Hemicellulose (arbinan)
GH43	Ep.00g033800.m01	PF04616.9	Endo-α-1,5-L-arabinanase	Hemicellulose (arbinan)
GH43	Ep.00g072460.m01	PF04616.9	Endo-α-1,5-L-arabinanase	Hemicellulose (arbinan)
GH43	Ep.00g017360.m01	PF04616.9	Endo-α-1,5-L-arabinanase	Hemicellulose (arbinan)
GH51	Ep.00g049020.m01	PF06964.7	α-L-arabinofuranosidase	Hemicellulose (arbinan)
GH54	Ep.00g038770.m01	PF09206.6	α-L-arabinofuranosidase	Hemicellulose (arbinan)
GH62	Ep.00g082120.m01	PF03664.8	α-L-arabinofuranosidase	Hemicellulose (arbinan)
GH10	Ep.00g006060.m01	PF00331.15	Endo-β-1,4-xylanase	Hemicellulose (arbinan)
GH10	Ep.00g084100.m01	PF12915.2	Endo-β-1,4-xylanase	Hemicellulose (arbinan)
GH10	Ep.00g062650.m01	PF00331.15	Endo-β-1,4-xylanase	Hemicellulose (arbinan)
GH11	Ep.00g028410.m01	PF00457.12	Endo-β-1,4-xylanase	Hemicellulose (arbinan)
GH11	Ep.00g025810.m01	PF00457.12	Endo-β-1,4-xylanase	Hemicellulose (arbinan)
GH12	Ep.00g082310.m01	PF01670.11	endo-xyloglucanase	Hemicellulose (arbinan)
GH12	Ep.00g067140.m01	PF01670.11	endo-xyloglucanase	Hemicellulose (arbinan)
CE1	Ep.00g065300.m01	PF00024.21	Acetylxylan esterase	Hemicellulose (arbinan)
CE1	Ep.00g014390.m01	PF00756.15	Acetylxylan esterase	Hemicellulose (arbinan)
CE2	Ep.00g034640.m01	PF13472.1	Acetylxylan esterase	Hemicellulose (arbinan)
GH93	Ep.00g013140.m01	PF13088.1	Exo-α-1,5-L-arabinofuranosidase	Hemicellulose (arabinanose)
GH142	Ep.00g065040.m01	PF06202.9	β-L-arabinofuranosidase	Hemicellulose (arabinanose)
GH146	Ep.00g090900.m01	PF07944.7	β-L-arabinofuranosidase	Hemicellulose (arabinanose)
GH146	Ep.00g035610.m01	PF07944.7	β-L-arabinofuranosidase	Hemicellulose (arabinanose)
AA5	Ep.00g066690.m01	PF07250	Galactose oxidase	Hemicellulose (galactose)
GH27	Ep.00g009190.m01	PF02065.13	α-galactosidase	Hemicellulose (galactose)
GH53	Ep.00g049270.m01	PF07745.8	endo-β-1,4-galactanase	Hemicellulose (galactose)
GH53	Ep.00g023560.m01	PF07745.8	endo-β-1,4-galactanase	Hemicellulose (galactose)
GH53	Ep.00g055170.m01	PF07745.8	endo-β-1,4-galactanase	Hemicellulose (galactose)
GH95	Ep.00g074320.m01	PF14498.1	α-L-galactosidase	Hemicellulose (galactose)
GH114	Ep.00g062230.m01	PF03537.8	endo-α-1,4-galactosaminidase	Hemicellulose (galactose)
GH115	Ep.00g071920.m01	PF03648.9	Xylan α-1,2-(4-O-methyl)-glucuronidase	Hemicellulose (galactose)
GH135	Ep.00g063890.m01	PF12138.3	endo-α-1,4-N-acetylgalactosaminidase	Hemicellulose (galactose)
GH6	Ep.00g027000.m01	PF00734.13	Endo-β-1,4-glucanase	Hemicellulose (glucans)
GH6	Ep.00g037220.m01	PF01341.12	Endo-β-1,4-glucanase	Hemicellulose (glucans)
GH7	Ep.00g074170.m01	PF00840.15	Endo-β-1,4-glucanase	Hemicellulose (glucans)
GH7	Ep.00g007280.m01	PF00840.15	Endo-β-1,4-glucanase	Hemicellulose (glucans)
GH16	Ep.00g045690.m01	PF00722.16	Endo-β-1,3-glucanase	Hemicellulose (glucans)
GH16	Ep.00g063100.m01	PF00722.16	Endo-β-1,3-glucanase	Hemicellulose (glucans)
GH16	Ep.00g078950.m01	PF00722.16	Endo-β-1,3-glucanase	Hemicellulose (glucans)
GH16	Ep.00g069650.m01	PF00722.16	Endo-β-1,3-glucanase	Hemicellulose (glucans)
GH16	Ep.00g032460.m01	PF00722.16	Endo-β-1,3-glucanase	Hemicellulose (glucans)
GH16	Ep.00g058160.m01	PF00722.16	Endo-β-1,3-glucanase	Hemicellulose (glucans)
GH17	Ep.00g008060.m01	PF00332.13	Endo-β-1,3-glucanase	Hemicellulose (glucans)
GH17	Ep.00g053040.m01	PF00332.13	Endo-β-1,3-glucanase	Hemicellulose (glucans)
GH17	Ep.00g065200.m01	PF00332.13	Endo-β-1,3-glucanase	Hemicellulose (glucans)
GH17	Ep.00g039010.m01	PF00332.13	Endo-β-1,3-glucanase	Hemicellulose (glucans)
GH45	Ep.00g068830.m01	PF02015.11	Endo-β-1,4-glucanase	Hemicellulose (glucans)
GH64	Ep.00g003160.m01	PF16483.8	Endo-β-1,3-glucanase	Hemicellulose (glucans)
GH81	Ep.00g082870.m01	PF10243.4	Endo-β-1,3-glucanase	Hemicellulose (glucans)
GH128	Ep.00g084710.m01	PF11790.3	Endo-β-1,3-glucanase	Hemicellulose (glucans)
GH128	Ep.00g012370.m01	PF11790.3	Endo-β-1,3-glucanase	Hemicellulose (glucans)
GH128	Ep.00g044710.m01	PF11790.3	Endo-β-1,3-glucanase	Hemicellulose (glucans)
GH132	Ep.00g092560.m01	PF03856.8	Exo-β-1,3-glucanase	Hemicellulose (glucans)
GH47	Ep.00g041820.m01	PF01532.15	Mannosyl-oligosaccharide α-1,2-mannosidase	Hemicellulose (mannose)
GH47	Ep.00g081180.m01	PF01532.15	Mannosyl-oligosaccharide α-1,2-mannosidase	Hemicellulose (mannose)
GH76	Ep.00g040900.m01	PF03663.9	α-1,6-mannanase	Hemicellulose (mannose)
GH76	Ep.00g079360.m01	PF03663.9	α-1,6-mannanase	Hemicellulose (mannose)
GH78	Ep.00g042190.m01	PF05592.6	α-L-rhamnosidase	Hemicellulose (mannose)
GH92	Ep.00g048360.m01	PF07971.7	α-1,4-mannosidase	Hemicellulose (mannose)
GH125	Ep.00g081770.m01	PF06824.6	exo-α-1,6-mannosidase	Hemicellulose (mannose)
GH125	Ep.00g060470.m01	PF06824.6	exo-α-1,6-mannosidase	Hemicellulose (mannose)
GH67	Ep.00g086010.m01	PF03648.9	Xylan α-1,2-(4-O-methyl)-glucuronidase	Hemicellulose (uronic acids)
GH79	Ep.00g044850.m01	PF06989.7	β-glucuronidase	Hemicellulose (uronic acids)
GH105	Ep.00g036690.m01	PF07470.8	D-4,5-unsaturated α-galacturonidase	Hemicellulose (uronic acids)
GH20	Ep.00g004780.m01	PF02838.10	β-N-acetylhexosaminidase	Hexosamine
AA1	Ep.00g003340.m01	PF07732	Multicopper oxidase	Lignin
AA1	Ep.00g086040.m01	PF07732	Multicopper oxidase	Lignin
AA1	Ep.00g062170.m01	PF07732	Multicopper oxidase	Lignin
AA1	Ep.00g032930.m01	PF07732	Multicopper oxidase	Lignin
AA2	Ep.00g062180.m01	PF00141	Peroxidase	Lignin
AA3	Ep.00g031600.m01	PF00732	Aryl alcohol oxidase	Lignin
AA3	Ep.00g041430.m01	PF00732	Aryl alcohol oxidase	Lignin
AA3	Ep.00g005910.m01	PF00732	Aryl alcohol oxidase	Lignin
AA3	Ep.00g046410.m01	PF00732	Aryl alcohol oxidase	Lignin
AA3	Ep.00g082320.m01	PF00732	Aryl alcohol oxidase	Lignin
AA3	Ep.00g046440.m01	PF00732	Aryl alcohol oxidase	Lignin
AA3	Ep.00g035860.m01	PF00732	Aryl alcohol oxidase	Lignin
AA3	Ep.00g041340.m01	PF00732	Aryl alcohol oxidase	Lignin
CE8	Ep.00g015010.m01	PF01095.14	Pectin methylesterase	Pectin
CE8	Ep.00g028320.m01	PF01095.14	Pectin methylesterase	Pectin
CE8	Ep.00g001980.m01	PF01095.14	Pectin methylesterase	Pectin
CE8	Ep.00g004170.m01	PF01095.14	Pectin methylesterase	Pectin
GH2	Ep.00g045820.m01	PF02837.13	β-galactosidase	Pectin
GH2	Ep.00g075560.m01	PF02837.13	β-galactosidase	Pectin
GH5	Ep.00g085260.m01	PF00150.13	Endo-β-1,6-galactanase	Pectin
GH5	Ep.00g060670.m01	PF00150.13	Endo-β-1,6-galactanase	Pectin
GH5	Ep.00g031200.m01	PF00150.13	Endo-β-1,6-galactanase	Pectin
GH5	Ep.00g037240.m01	PF00150.13	Endo-β-1,6-galactanase	Pectin
GH5	Ep.00g005540.m01	PF00150.13	Endo-β-1,6-galactanase	Pectin
GH5	Ep.00g074330.m01	PF00150.13	Endo-β-1,6-galactanase	Pectin
GH5	Ep.00g014300.m01	PF00150.13	Endo-β-1,6-galactanase	Pectin
GH5	Ep.00g081230.m01	PF00150.13	Endo-β-1,6-galactanase	Pectin
GH5	Ep.00g072670.m01	PF00150.13	Endo-β-1,6-galactanase	Pectin
GH5	Ep.00g065870.m01	PF00150.13	Endo-β-1,6-galactanase	Pectin
GH28	Ep.00g048380.m01	PF00295.12	Endo-polygalacturonase	Pectin
GH28	Ep.00g009600.m01	PF00295.12	Endo-polygalacturonase	Pectin
GH28	Ep.00g074850.m01	PF00295.12	Endo-polygalacturonase	Pectin
GH28	Ep.00g084490.m01	PF00295.12	Endo-polygalacturonase	Pectin
GH28	Ep.00g079230.m01	PF00295.12	Endo-polygalacturonase	Pectin
GH28	Ep.00g026980.m01	PF00295.12	Endo-polygalacturonase	Pectin
GH28	Ep.00g021250.m01	PF00295.12	Endo-polygalacturonase	Pectin
GH35	Ep.00g023860.m01	PF01301.14	β-galactosidase	Pectin
GH35	Ep.00g035150.m01	PF01301.14	β-galactosidase	Pectin
PL1	Ep.00g028120.m01	PF00544.14	Pectate lyase	Pectin
PL1	Ep.00g092740.m01	PF00544.14	Pectate lyase	Pectin
PL1	Ep.00g073980.m01	PF00544.14	Pectate lyase	Pectin
PL1	Ep.00g037200.m01	PF00544.14	Pectate lyase	Pectin
PL3	Ep.00g027020.m01	PF03211.8	Pectate lyase	Pectin
PL3	Ep.00g057340.m01	PF12708.2	Pectate lyase	Pectin
PL3	Ep.00g038030.m01	PF12708.2	Pectate lyase	Pectin
PL3	Ep.00g050710.m01	PF12708.2	Pectate lyase	Pectin
PL3	Ep.00g036650.m01	PF03211.8	Pectate lyase	Pectin
PL3	Ep.00g026880.m01	PF03211.8	Pectate lyase	Pectin
PL3	Ep.00g005980.m01	PF03211.8	Pectate lyase	Pectin
PL4	Ep.00g057920.m01	PF14686.1	Rhamnogalacturonan lyase	Pectin
PL4	Ep.00g092400.m01	PF14686.1	Rhamnogalacturonan lyase	Pectin
PL4	Ep.00g011470.m01	PF14686.1	Rhamnogalacturonan lyase	Pectin
PL4	Ep.00g070450.m01	PF14686.1	Rhamnogalacturonan lyase	Pectin
GH13	Ep.00g046360.m01	PF00128.19	Alpha amylase	Starch
GH13	Ep.00g010930.m01	PF00128.19	Alpha amylase	Starch
GH15	Ep.00g046350.m01	PF00723.16	Glucoamylase	Starch

### 3.4. Carbohydrate-active enzymes

**Figure 4.**
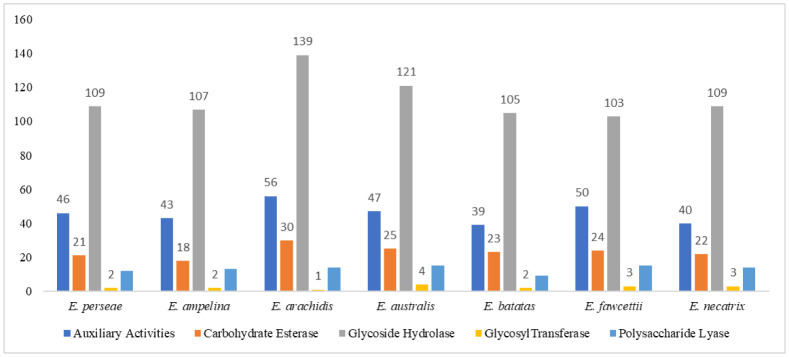
Distribution of CAZymes in seven species of *Elsinoe*. The numbers above the bar indicate the number of proteins in each group.

Carbohydrate-active enzymes, known as CAZymes, are a broad category of enzymes that participate in the synthesis and degradation of carbohydrates and glycoconjugates [53],[54]. These enzymes are classified into six categories: glycoside hydrolases (GH), polysaccharide lyases (PL), carbohydrate esterases (CE), auxiliary activity (AA), glycosyltransferases (GT), and carbohydrate-binding modules (CBM) [46]. To determine the CAZymes in *E. perseae* and its closely related species, data from multiple sources was used, such as the blast description, gene ontology, EC number, PFAM domain, and the annotation results from the CAZy database [46]. A total of 190 CAZymes were detected in the secretome of *E. perseae*, as shown in Figure 4, Table S3, and Table S4. The CAZymes constituted 29.3% of the secretome of *E. perseae*, which was distributed in 67 CAZymes families (Table S3, Table S4). The number of CAZymes among the closely related species range from 178 to 240, with the lowest count observed in *E. batatas* and the greatest count observed in *E. australis* (Figure 4). Glycosidase hydrolase was found to be prominent among CAZymes in all species (Figure 4). In total, 78 CAZymes families were identified among all the seven species of *Elsinoe*, of which 51 were shared by all species. These families include 8 AA, 6 CE, 33 GH, 2 GT, and 3 PL families (Tables S3 and S4). Certain CAZymes exhibited restricted distribution, such as GH72 and GH81, which were exclusively detected in *E. batatas* and *E. perseae*, respectively (Tables S3 and S4). GH132 is exclusively present in *E. perseae* and *E. australis*; GH134 is exclusively found in *E. arachidis* and *E. batatas*; and GH135 is exclusively present in *E. perseae* and *E. arachidis* (Tables S3 and S4).

### 3.5. Cell wall–degrading enzymes

The cell wall of the avocado is composed of various polysaccharides, including cellulose, hemicellulose, and pectin, which provide structural integrity and protection to the cells [55],[56]. To overcome these physical barriers, *E. persea* may secrete cell wall–degrading enzymes, such as cellulases and pectinases, to weaken and disrupt the structural integrity of the cell wall components. With the exception of the GT family, the GH, CE, PL, and AA families of CAZymes are classified as cell wall–degrading enzymes due to their involvement in the decomposition of several plant cell wall components [57],[58]. Cellulose is made up of a straight chain of numerous β-linked D-glucose units. The enzymes responsible for breaking down cellulose include exo-β-1,4-glucanases, endo-β-1,4-glucanases, β-1,4-glucosidases, cellobiose dehydrogenase, and lytic cellulose monooxygenase [59]–[61]. Out of the 190 CAZymes of *E. perseae*, 46 were identified as having the ability to break down cellulose. These include 8 glucooligosaccharide oxidases, 13 β-glucosidases, 1 α-glucosidase, 6 cellobiose dehydrogenases, and 18 lytic cellulose monooxygenases (Table 2). One α-glucosidase was identified within the GH31 family, while eight glucooligosaccharide oxidases were only discovered in the AA7 families (Table 2). A total of 18 lytic cellulose monooxygenases were identified in the AA9 (15) and AA16 (3) families, along with six cellobiose dehydrogenase enzymes in the AA3 (3) and AA8 (3) families (Table 2). Thirteen β-glucosidase enzymes were classified into four CAZymes families: GH1 (2), GH3 (8), GH39 (1), and GH131 (2) (Table 2).

Hemicellulose is a significant constituent of the plant cell wall, comprising xyloglucans, xylans, mannans, glucomannans, and beta-(1-->3,1-->4)-glucans [62]. The primary enzymes responsible for breaking down hemicellulose are L-arabinanases, D-galactanases, D-mannanases, and D-xylanases [63]. In addition, numerous fungal species have been found to possess endo-β-1,4-glucanase that exhibit xyloglucanase activity [64]. Among the 190 CAZymes, 64 proteins were found to have the capability of degrading hemicellulose (Table 2). This group consisted of 60 GH proteins, 3 CE proteins, and 1 AA protein. The GH group consisted of 10 members from GH43, 6 members from GH16, 4 members from GH17, 3 members each from GH10, GH53, and GH128, 2 members each from GH11, GH12, GH47, GH76, GH125, and GH146, and one member each from 19 GH families (GH105, GH114, GH115, GH132, GH135, GH142, GH20, GH27, GH51, GH54, GH62, GH64, GH67, GH78, GH79, GH81, GH92, GH93, and GH95) and the AA5 family (Table 2). The predominant hemicellulose-degrading enzyme found in the secretome of *E. perseae* was endo-β-1,3-glucanase (15), which selectively breaks down chains of glucans. This enzyme is found in GH16 (6), GH17 (4), GH128 (3), GH64 (1), and GH81 (1). Interestingly, the GH81 enzyme was found only in *E. perseae*, suggesting a promising candidate for future research to understand the pathogenicity of *E. perseae* on avocado. It was also found that the secretome has 10 endo-α-1,5-L-arabinanase enzymes belonging to the CAZyme GH43, 3 α-L-arabinofuranosidase enzymes (GH51 & GH54), 3 β-L-arabinofuranosidase enzymes (GH142 & GH146), and 1 exo-α-1,5-L-arabinofuranosidase enzyme (GH93). These enzymes play a role in breaking down polysaccharides composed of arabinan molecules [65]. In addition, the secretome of *E. perseae* was revealed to have 27 glycosidic hydrolases that specifically break down the polysaccharide composed of xylan (7), galactose (8), mannose (8), and uronic acids (4) (Table 2) [66]–[69]. Furthermore, the secretome also contained three esterases that degrade hemicellulose, as indicated in Table 2.

Pectin is a significant constituent of the primary cell walls found in all terrestrial plants. It consists of several polysaccharides that are rich in galacturonic acid [70]. All primary cell walls are believed to include three primary pectic polysaccharides: homogalacturonan, rhamnogalacturonan-I, and rhamnogalacturonan-II [71],[72]. The secretome of *E. perseae* contains 40 pectin-degrading enzymes, which are distributed among 11 CAZymes (Table 2). These include 10 endo-β-1,6-galactanase (GH5), 7 endo-polygalacturonase (GH28), 4 pectin methylesterase (CE8), 11 pectate lyase (PL1 and 3), 4 rhamnogalacturonan lyase (PL4), and 4 β-galactosidase (GH2 and 35) (Table 2). Furthermore, individuals belonging to auxiliary activity (AA) families were discovered to possess the capability to break down lignin (Table 2). This category consists of four AA1 multicopper oxidases, one AA2 peroxidase, and eight AA2 aryl alcohol oxidases (Table 2). Furthermore, the refined secretome includes 13 cutinases that break down cutin as well as three amylases that break down starch (Table 2). The role of cutinases in pathogenicity has been demonstrated for many phytopathogenic fungi [73].

### 3.6. Secreted proteases

**Figure 5.**
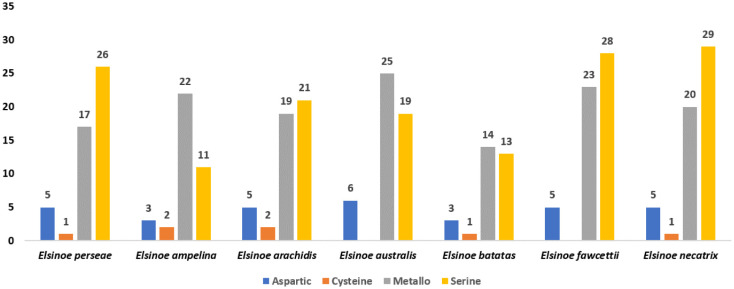
Secretory proteases in *Elsinoe species*. The numbers above the bar indicate the number of proteins in each group.

Multiple studies have demonstrated that plant pathogenic fungi secrete proteases that break down plant antimicrobial proteins, as well as protease inhibitors (PIs) to enhance their ability to cause disease [74]. The BlastP search conducted on the MEROPS database yielded the discovery of 49 potential proteases from the 654 refined secretome (Figure 5). These proteases were categorized into various categories based on their catalytic residues, as shown in Table S4. Serine proteases were the most prevalent among the proteases, with a total of 26. They were followed by metalloproteases (17), aspartic proteases (5), and carboxy proteases (1) (Table S4). The serine proteases encompassed families S8, S9, S10, S28, S41, and S51. Among them, the S8 family was identified as the most prevalent (Table S4). The categorization of metalloprotease members was based on their resemblance to the established members of the M6, M20, M28, and M36 families (Table S4). The number of proteases varies among closely related species, ranging from 31 in *E. batatas* to 56 in *E. fawcettii* and *E. necatrix* (Figure 5; Table S5). Comparative analysis showed that serine protease was predominant in *E. perseae*, *E. necatrix*, *E. arachidis*, and *E. fawcettii*, while metalloprotease was predominant in the remaining species (Figure 5).

### 3.7. Putative effector proteins

**Figure 6.**
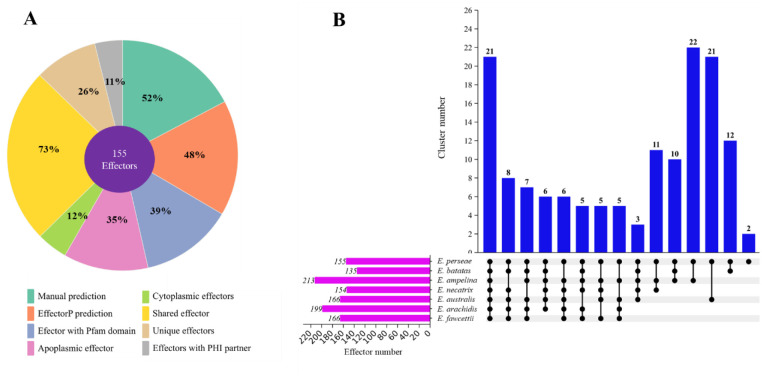
Effectors in *E. perseae*. A. Details of effectors in *E. perseae* B. Orthologue analysis of putative effectors in seven species of *E. perseae*. Clusters of orthologous genes were detected utilizing Orthovenn3 and displayed utilizing UpsetR. For the analysis, the e-value cutoff of 1 × 10^−10^ was utilized. Blue bars represent the number of clusters shared by *Elsinoe* species, represented by numbers shown above the bar. Line and dot connections between species that share orthologs.

The combination of EffectorP and manual examination was used to select probable effector proteins with the following features: a signal peptide for secretion, absence of trans-membrane domains, relatively modest size, and high cysteine content [6],[75]. The study led to the discovery of 155 proteins as potential "effector" candidates (Figure 6A, Table S6). Effectors ranged in length from 88 to 395 aa (Table S6). Among these candidates, 43 (27.7%) were between 100 and 200 amino acids long, 56 (36.1%) were between 200 and 300 amino acids long, and 52 (33.5%) were between 300 and 400 amino acids long (Table S6). Four candidates (Ep.00g073550.m01, Ep.00g033060.m01, Ep.00g017120.m01, and Ep.00g059420.m01) were identified as having a length of less than 100 aa (Table S6). The identified effectors exhibited a range of cysteine residues, varying from 2 to 23. Among these putative effectors, 74.8% (116) had more than four cysteine residues, as shown in Table S6. Out of the total 155 potential effectors, EffectorP identified 55 as apoplastic effectors and 20 as cytoplasmic effectors (Figure 6A, Table S6). Of the 155 potential effectors, 80 proteins were selected manually as “putative effectors” based on their small size and number of cysteine residues (Figure 6A, Table S6). Of the 155 putative effector proteins, functional domains were found in 60 proteins (Figure 6A, Table S6). Of these, six apoplastic effectors (Ep.00g005690.m01, Ep.00g005690.m01, Ep.00g005750.m01, Ep.00g005790.m01, Ep.00g033060.m01, and Ep.00g085650.m01) possessed the fungal hydrophobin domain (PF01185) (Table S6). Four potential apoplastic effectors (Ep.00g010150.m01, Ep.00g027650.m01, Ep.00g072050.m01, and Ep.00g064760.m01) were found to have the LysM domain (PF01476) (Table S6). The pathogen effector domain known as putative necrosis-inducing factor (PF14856) was detected in three apoplastic putative effectors (Ep.00g021750.m01, Ep.00g067220.m01, and Ep.00g091030.m01). Additionally, the necrosis-inducing protein (NPP1) domain (PF05630) was observed in one protein (Ep.00g018600.m01) (Table S6). Orthologue analysis revealed that *E. perseae* had 118 effector clusters, of which 116 were shared among closely related species and 2 were in paralogs (Figure 6B). The inparalogs (Ep.00g005790.m01, Ep.00g005750.m01, Ep.00g018330.m01, and Ep.g018320.m01) exhibited over 90% sequence similarity, suggesting a recent duplication event. Remarkably, a total of 40 effectors were identified to be specific to *E. perseae* and do not share any similarity with proteins reported in other related species (Table S6). An examination of the functionality of these effectors may provide further understanding of the pathogenic nature of *E. perseae* on avocado. Notably, the research revealed that *E. australis* and *E. batatas* had 40 and 20 distinct protein clusters, respectively (not shown in this study). Examining these proteins could uncover the underlying biological mechanism responsible for scab disease in their specific host plants.

### 3.8. Putative virulence factors in E. perseae

In order to find the homologs of the *E. perseae* secretory proteins that are linked to pathogenicity in other phytopathogens, we examined all secretory proteins in *E. perseae*, including all the potential effectors, against the PHI (Pathogen Host Interactions) database [49]. The protein sequences in the PHI database are categorized based on the outcomes of mutation experiments. These categories include, for instance, loss of pathogenicity, unaffected pathogenicity, reduced virulence, increased virulence, and effector (plant avirulence determinant). For instance, the "loss of pathogenicity" group comprises proteins that, when present in mutant strains, result in failure to induce disease in the host as compared to the natural type. According to the PHI annotation, out of the 654 secretome, 104 had PHI homologues, which included 61 CAZymes, 25 proteases, and 18 putative effectors (Table S7). Out of the 61 CAZymes, 26 were classified as "reduced virulence". These included 8 enzymes that break down cellulose (5 β-glucosidase, 1 cellobiose dehydrogenase, 1 lytic cellulose monooxygenase, and 1 α-glucosidase), 9 enzymes that break down hemicellulose (4 endo-β-1,4-xylanases, 2 endo-β-1,3-glucanases, 1 endo-β-1,4-glucanase, and 2 exo-α-1,6-mannosidases), 8 enzymes that break down pectin (3 endo-β-1,6-galactanases and 5 pectate lyases), and 1 enzyme that breaks down lignin, specifically encoding an aryl alcohol oxidase (Table S7). The counterparts of these enzymes were documented to contribute to the virulence of many phytopathogenic fungi. For example, endo-β-1,4-xylanase has been demonstrated to play a role in the pathogenicity of certain fungal diseases, such as *Verticillium dahlia*, *Ustilago maydis*, and *Valsa mali* [76],[77].

Furthermore, according to the PHI database, two CAZymes were identified as "effector_(plant_avirulence_determinant)", specifically two lytic cellulose monooxygenases. Chen et al. demonstrated that the homolog of lytic cellulose monooxygenase in *Magnaporthe oryzae* (MoCDIP) triggered cellular apoptosis upon expression in rice plant cells [78]. Furthermore, a *Podosphaera xanthii* gene called PHEC27213, which encodes a lytic cellulose monooxygenase, was found to inhibit the immune response triggered by chitin in the cucurbit host [79]. Out of the 61 CAZymes, four were categorized as "increased virulence (hypervirulence)", and 29 were categorized as "unaffected pathogenicity" (Table S7). Out of the 25 proteases with PHI partners, 1 cysteine protease was designated as "loss of pathogenicity", 1 was designated as "effector_(plant_avirulence_determinant)", and 11 were designated as "reduced virulence" (Table S7). Furthermore, two proteases were designated as "unaffected pathogenicity" according to the PHI annotation (Table S7). Among the putative effectors, we discovered PHI partners for 18 proteins, 5 of which were designated as "effector_(plant_avirulence_determinant)". Among them, two proteins (Ep.00g072050.m01, Ep.00g010150.m01) include the LysM domain, whereas one protein (Ep.00g027520.m01) contains the cerato-platanin domain (Table S7). Multiple studies have demonstrated that proteins containing the LysM domain operate as virulence factors in many phytopathogenic fungi by inhibiting the immune response triggered by chitin in host plants [80],[81]. Cerato-platanins are a collection of small proteins that are rich in cysteine and are released by certain plant pathogenic fungi to facilitate virulence on the host plant [82]. Three potential effectors were classified as "reduced virulence" (Table S7). Among these, two genes encode a fungal hydrophobin and a peroxidase. These proteins' homologs have been demonstrated to be essential for the pathogenicity of numerous fungal infections [83].

## 4. Conclusions

The present study employed a bioinformatics pipeline to thoroughly describe the putative secretory proteins of *E. perseae*. A total of 190 carbohydrate-active enzymes (CAZymes), 90 proteases, and 155 potential effector proteins were detected in the secretome. The investigation showed that *E. perseae* had multiple families of enzymes capable of breaking down cellulose, hemicellulose, pectin, and lignin, as well as numerous proteases to defeat the initial defense mechanisms of plants. In addition, the comparative study showed that *E. perseae* had 41 putative effectors that were specific to its species, as well as many putative virulence factors. The present work will serve as a potential resource for research focused on comprehending the pathogenicity mechanism in the interaction between *E. perseae* and avocado.

## Data availability statement

The datasets analyzed in this study can be found in online repositories. The names of the repositories and accession numbers can be found in the article (Table 1). Additional data generated in this study can be found in the supplementary material.

## Funding

The author extends his appreciation to the Deanship of Scientific Research, Vice Presidency for Graduate Studies and Scientific Research, King Faisal University, Saudi Arabia, for funding this research work (Project number KFU242183).

## Use of AI tools declaration

The authors declare they have not used Artificial Intelligence (AI) tools in the creation of this article.










