# Supplementary material for: Comparative secretome analysis unveils species-specific virulence factors in Elsinoe perseae, the causative agent of the scab disease of avocado (Persea americana)
Source: AIMS Microbiol. 2024 Oct 28;10(4):894–916. doi: 10.3934/microbiol.2024039 (PMC11609419; doi:10.3934/microbiol.2024039)
Supplement: Supplementary file 1 [file microbiol-10-04-039-s001.pdf]

AIMS Microbiology

AIMS Microbiology, 10(4): 894–916.
DOI: 10.3934/microbiol.2024039
Received: 11 July 2024
Revised: 17 October 2024
Accepted: 21 October 2024
Published: 28 October 2024

http://www.aimspress.com/journal/microbiology

Research article

Comparative secretome analysis unveils species-specific virulence factors in *Elsinoe perseae*, the causative agent of the scab disease of avocado (*Persea americana*)

Biju Vadakkemukadiyil Chellappan

Department of Biological Sciences, College of Science, King Faisal University, P.O. Box 420, Al-Ahsa 31982, Saudi Arabia

* **Correspondence:** Email: bchellappan@kfu.edu.sa

Table S2. Secretome clusters in *Elsinoe* species.

Species	Secretome	clusters	singletons
Elsinoe arachidis	781	680	80
Elsinoe australis	676	559	112
Elsinoe fawcettii	658	584	65
Elsinoe perseae	654	879	75
Elsinoe ampelina	621	538	74
Elsinoe necatrix	619	552	60
Elsinoe batatas	569	523	38

Table S4. Secretory proteases in *E. perseae*.

Protein id	Protease family	Pfam domain	Protease name
Ep.00g035870.m01	Asp	PF00026.26	Eukaryotic aspartyl protease
Ep.00g020820.m01	Asp	PF00026.26	Eukaryotic aspartyl protease
Ep.00g067570.m01	Asp	PF00026.26	Eukaryotic aspartyl protease
Ep.00g076280.m01	Asp	PF00026.26	Eukaryotic aspartyl protease
Ep.00g017800.m01	Asp	PF00026.26	Eukaryotic aspartyl protease
Ep.00g045610.m01	Peptidase_C13	PF01650.21	Peptidase C13 family
Ep.00g004710.m01	Peptidase_M14	PF00246.27	Zinc carboxypeptidase
Ep.00g024300.m01	Peptidase_M14	PF00246.27	Zinc carboxypeptidase
Ep.00g016040.m01	Peptidase_M14	PF00246.27	Zinc carboxypeptidase
Ep.00g092320.m01	Peptidase_M14	PF00246.27	Zinc carboxypeptidase
Ep.00g035780.m01	Peptidase_M14	PF00246.27	Zinc carboxypeptidase
Ep.00g091440.m01	Peptidase_M14	PF00246.27	Zinc carboxypeptidase
Ep.00g005040.m01	Peptidase_M14	PF00246.27	Zinc carboxypeptidase
Ep.00g057470.m01	Peptidase_M14	PF00246.27	Zinc carboxypeptidase
Ep.00g059880.m01	Peptidase_M20	PF01546.31	Peptidase family M20/M25/M40
Ep.00g058070.m01	Peptidase_M20	PF01546.31	Peptidase family M20/M25/M40
Ep.00g074190.m01	Peptidase_M20	PF01546.31	Peptidase family M20/M25/M40
Ep.00g052080.m01	Peptidase_M20	PF01546.31	Peptidase family M20/M25/M40
Ep.00g001270.m01	Peptidase_M28	PF04389.20	Peptidase family M28
Ep.00g058100.m01	Peptidase_M36	PF02128.18	Fungalysin metallopeptidase (M36)
Ep.00g021270.m01	Peptidase_M36	PF02128.18	Fungalysin metallopeptidase (M36)
Ep.00g005770.m01	Peptidase_M36	PF02128.18	Fungalysin metallopeptidase (M36)
Ep.00g091530.m01	Peptidase_M6	PF05547.14	Immune inhibitor A peptidase M6
Ep.00g044050.m01	Peptidase_S10	PF13933.9	Putative peptidase family
Ep.00g079410.m01	Peptidase_S10	PF12146.11	Serine aminopeptidase
Ep.00g035350.m01	Peptidase_S10	PF12146.11	Serine aminopeptidase
Ep.00g038690.m01	Peptidase_S10	PF00450.25	Serine carboxypeptidase
Ep.00g046060.m01	Peptidase_S10	PF00450.25	Serine carboxypeptidase
Ep.00g008670.m01	Peptidase_S10	PF00450.25	Serine carboxypeptidase
Ep.00g044070.m01	Peptidase_S10	PF00450.25	Serine carboxypeptidase
Ep.00g058640.m01	Peptidase_S10	PF00450.25	Serine carboxypeptidase
Ep.00g028720.m01	Peptidase_S28	PF05577.15	Serine carboxypeptidase S28
Ep.00g042350.m01	Peptidase_S28	PF05577.15	Serine carboxypeptidase S28
Ep.00g058970.m01	Peptidase_S41	PF03572.21	Peptidase family S41
Ep.00g057770.m01	Peptidase_S41	PF03572.21	Peptidase family S41
Ep.00g089810.m01	Peptidase_S51	PF03575.20	Peptidase family S51
Ep.00g092290.m01	Peptidase_S8	PF00082.25	Subtilase family
Ep.00g079090.m01	Peptidase_S8	PF00082.25	Subtilase family
Ep.00g048410.m01	Peptidase_S8	PF00082.25	Subtilase family
Ep.00g053640.m01	Peptidase_S8	PF00082.25	Subtilase family
Ep.00g085580.m01	Peptidase_S8	PF00082.25	Subtilase family
Ep.00g027270.m01	Peptidase_S8	PF00082.25	Subtilase family
Ep.00g091480.m01	Peptidase_S8	PF00082.25	Subtilase family
Ep.00g079870.m01	Peptidase_S8	PF00082.25	Subtilase family
Ep.00g008550.m01	Peptidase_S8	PF00082.25	Subtilase family
Ep.00g006840.m01	Peptidase_S8	PF00082.25	Subtilase family

Continued on next page

Protein id	Protease family	Pfam domain	Protease name
Ep.00g085180.m01	Peptidase_S9	PF00326.24	Prolyl oligopeptidase family
Ep.00g023460.m01	Peptidase_S9	PF00326.24	Prolyl oligopeptidase family
Ep.00g014390.m01	Peptidase_S9	PF00326.24	Prolyl oligopeptidase family

Table S5. Distribution of Secretory proteases in Elsinoe species.

class	E. perseae	E. ampelina	E. arachidis	E. australis	E. batatas	E. fawcetii	E. nextraxix
Peptidase_Asp	5	3	5	6	3	5	5
Peptidase_C13	1	1	1		1		1
Peptidase_C15		1	1				
Peptidase_C41							1
Peptidase_M4		1		6	1	2	2
Peptidase_M10			2				
Peptidase_M14	8	2	7	6	4	7	7
Peptidase_M19		9		1			
Peptidase_M20	4		5	1	3	6	5
Peptidase_M28	1	1		7	1	1	1
Peptidase_M28		7		1	1	2	
Peptidase_M36	3	1	4	2	2	1	1
Peptidase_M49					1		
Peptidase_M57							1
Peptidase_M6	1	1	1	1	1	1	1
Peptidase_M60						1	1
Peptidase_M66						2	1
Peptidase_S8	10		10				
Peptidase_S9	3	1	1	3	3	8	9
Peptidase_S10	8	8	6	11	7	17	14
Peptidase_S28	2	1	2	1	2	1	2
Peptidase_S33				1			
Peptidase_S41	2		1	2	1	1	2
Peptidase_S49							2
Peptidase_S51	1	1	1	1		1	

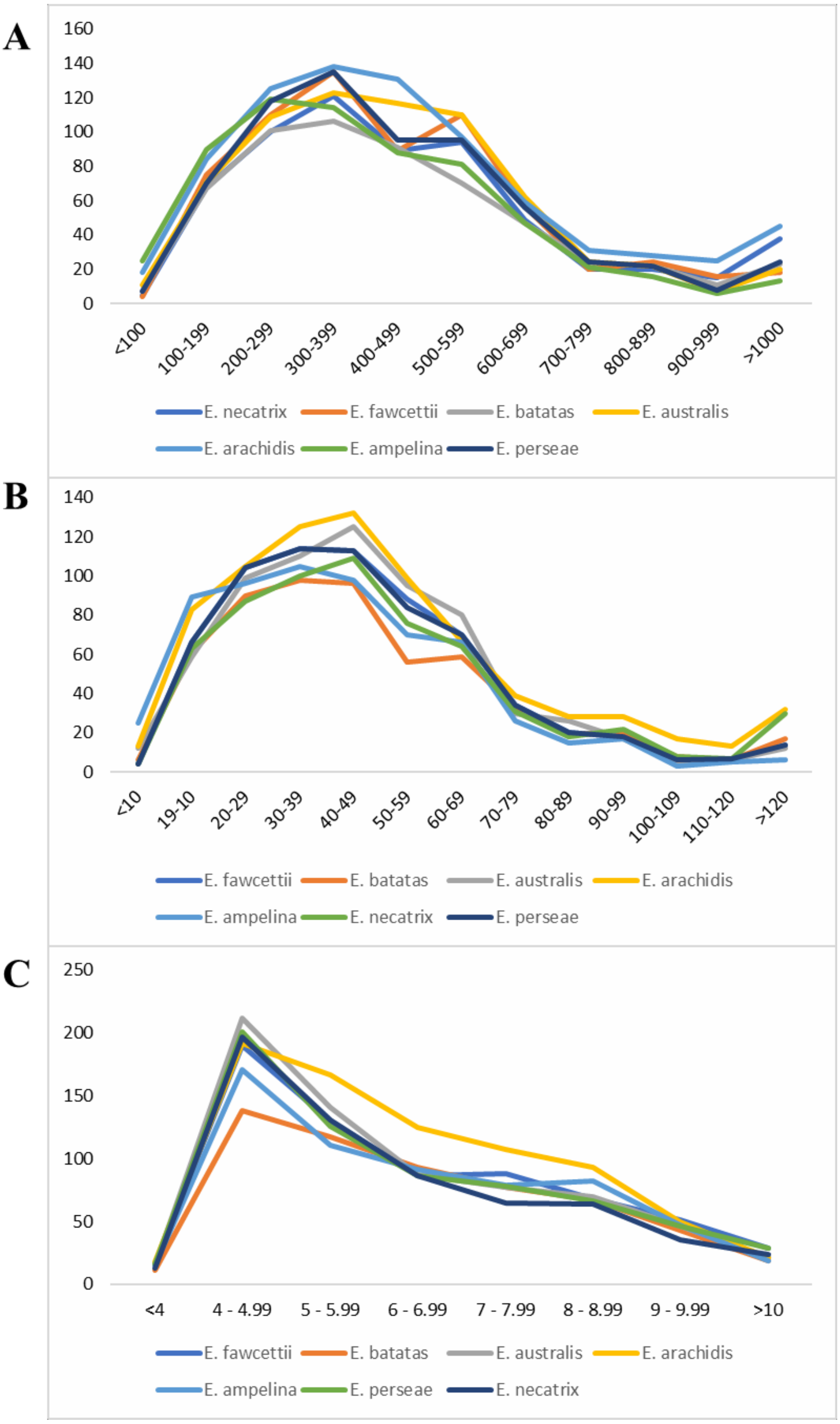

Figure S1. Characteristics of Secretome of *Elsinoe* species. A. Length distribution of the secretome B. Molecular weight of the secretome C. Theoretical PI of the secretome.

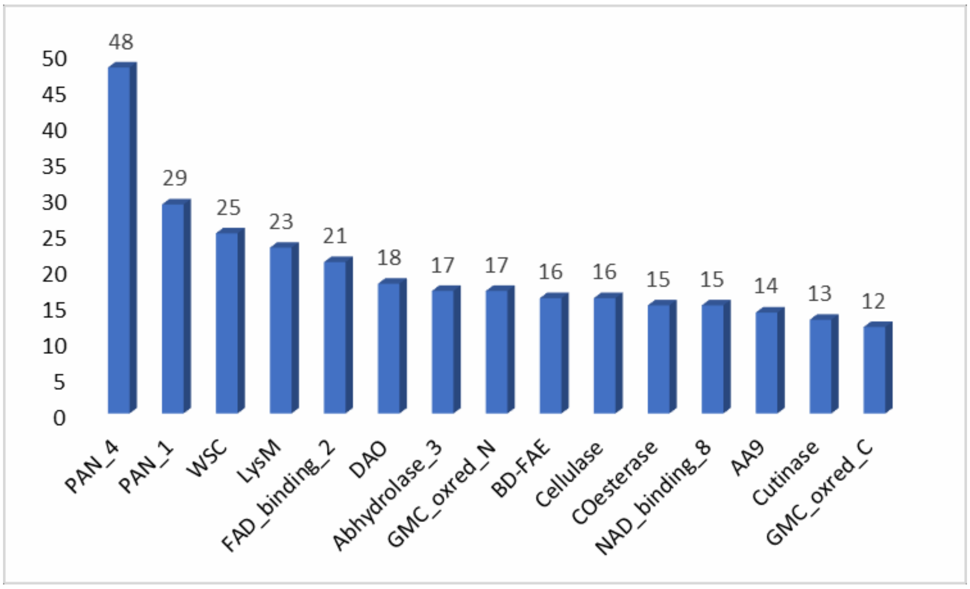

Figure S2. Domain distribution of secretome of *E. perseae*. The number above the bar represents the number of proteins harbour the respective domains.

AIMS Press

© 2024 the Author(s), licensee AIMS Press. This is an open access article distributed under the terms of the Creative Commons Attribution License (http://creativecommons.org/licenses/by/4.0)
